# Sanitary Era

**Published:** 1891-02

**Authors:** 


THE SANITARY ERA OR PROGRESSIVE HEALTH JOURNAL. Published on the 25th of each month by Wm. C. Conant, P. O. Box 3059, New York City. Subscription one dollar per year.

This is a quarto journal of about sixteen pages, each page having three columns. It is devoted to Sanitary Science, as its name indicates.

It is not intended for sanitarians only, but for citizens in general, for mothers, nurses, health-officers, etc. The copy before us, being No. 3 of Vol. V, contains a lot of very interesting matter upon water purifying, upon the theory of organic infection, upon Bacteriology, etc. W. M. J.

